# Acute pulmonary edema in an obstetric intensive care unit: A case series study

**DOI:** 10.1097/MD.0000000000011508

**Published:** 2018-07-13

**Authors:** Ana Carolina B. Pordeus, Leila Katz, Mariana C. Soares, Sabina B. Maia, Melania M. R. Amorim

**Affiliations:** a Postgraduate Program on Maternal and Child Health, Instituto de Medicina Integral Prof. Fernando Figueira; b Faculdade Pernambucana de Saúde (FPS), Recife, Pernambuco, Brazil.

**Keywords:** complications of pregnancy, echocardiography, intensive care unit, obstetrics, pulmonary edema

## Abstract

Acute pulmonary edema (PE) affects 0.08% to 1.5% of women during pregnancy and in the postpartum. At the *Instituto de Medicina Integral Prof. Fernando Figueira* (IMIP), acute PE accounts for 1.5% of admissions to the obstetric intensive care unit (ICU) and occurs in 9.3% of the patients admitted with *near miss* criteria. This study was conducted to describe the clinical/epidemiological profile of patients with acute PE in IMIP's obstetric ICU.

A case series of 50 patients with acute PE in an obstetric ICU in northeastern Brazil between August 2012 and March 2015. Frequency distribution and measures of central tendency/dispersion were calculated using Epi Info, version 7.1.5.

The mean age of the women was 27.2 years; 60% were from Recife; 50% had 8 to 11 years of schooling; 54.0% were primigravidas. Acute PE occurred antepartum (58%), postpartum (38%), or intrapartum (4.0%). Overall, 8% had had previous episodes; 6% relapsed during hospitalization; 4% died. Caesarean sections were common (78.0%), with 73.3% delivering at <37 weeks and 39.0% at <34 weeks. Etiologies were hypertensive (62%), cardiogenic (16.0%), both hypertensive and cardiogenic (20.0%) or due to fluid overload (2.0%). Irrespective of etiology, in the 24 hours preceding acute PE, fluid overload was present in 34.0%. Median time from diagnosis until resuscitation maneuvers was 5 minutes (within 30 minutes of diagnosis in 75.0% of patients). Mean ICU time was 5 days and mean hospitalization time 11 days.

Acute PE is a severe disease resulting in high maternal/perinatal morbidity/mortality rates. Most commonly, it occurred antepartum and associated with hypertension. Fluid overload appears to constitute an important trigger.

## 1 Introduction

Acute pulmonary edema (PE) affects 0.08% to 1.5% of women during pregnancy and in the postpartum.^[1,2]^ Preeclampsia/eclampsia is a major obstetric cause of acute PE,^[3]^ with 0.6% to 5% of patients with preeclampsia/eclampsia developing acute PE.^[4,5]^ At the *Instituto de Medicina Integral Prof. Fernando Figueira* (IMIP), acute PE accounts for 1.5% of admissions to the obstetric intensive care unit (ICU) and occurs in 9.3% of the patients admitted with *near miss* criteria.^[2,5]^

Decompensation resulting from prior cardiovascular disease may account for 17.8 cases of PE per 1000 hospitalizations antepartum and 6.3 cases per 1000 hospitalizations postpartum.^[6]^ In Brazil, the most prevalent form of cardiovascular disease in pregnancy is rheumatic valve disease.^[7]^ Fluid overload remains an important cause of acute PE when fluid balance is >2000 mL, increasing hydrostatic pressure.^[8]^ PE has been strongly associated with the infusion of fluids in women submitted to induced labor, Caesarean section, or prophylaxis with magnesium sulfate.^[9]^

Diagnosis of acute PE is clinical; nevertheless, the etiology of the condition is sometimes unclear. Additional tools such as Doppler echocardiography permit systolic and diastolic evaluations to be made, differentiating cases of cardiogenic from those of noncardiogenic origin.^[10]^ Lack of an accurate etiological diagnosis may delay initiation of specific treatment, potentially affecting maternal/perinatal outcomes.^[3]^

This study was conducted to describe the clinical/epidemiological profile of patients with acute PE in IMIP's obstetric ICU.

## 2 Materials and methods

### 2.1 Study design and population

This was a descriptive case series of patients seen in IMIP's obstetric ICU in Recife, the capital of Pernambuco, Brazil. The sample was consecutive, with participants being selected in 2 steps: *Retrospective phase*: the ICU's electronic database was reviewed to identify women diagnosed with acute PE between August 2012 and July 2014; *Prospective phase*: all women admitted to the unit with acute PE between August 2014 and March 2015 were invited to participate in the study and those who agreed and signed an informed consent form were enrolled. Women identified retrospectively, but whose diagnosis of acute PE was not confirmed after their charts were reviewed, were excluded from the study. Patients whose pregnancy was ectopic or who had gestational trophoblastic disease were also excluded.

Acute PE was defined as an acute respiratory event occurring in pregnancy or within 45 days of delivery, diagnosed by the presence of sudden-onset dyspnea, crackling rales, and/or decreased saturation.^[11]^ All cases were diagnosed and treated in compliance with this institute's guidelines, including the routine use of Doppler echocardiography for diagnostic evaluation.

### 2.2 Outcomes evaluated

The variables analyzed were age, schooling, place of residence, obstetric characteristics, principal signs/symptoms, resuscitation maneuvers (time between diagnosis and furosemide use), echocardiography results, type and duration of mechanical ventilation, anesthesia during delivery, number of hospitalization days and days in ICU, the moment acute PE occurred (pre/post/intrapartum), etiology, *near miss* criteria according to the 2010 World Health Organization (WHO) guidelines,^[12]^ and perinatal/neonatal outcomes.

### 2.3 Statistical analysis

Statistical analysis was conducted using Epi Info, version 7.1.5. Frequency distribution was used to describe the categorical variables, with measures of central tendency and dispersion being used to describe the numerical variables.

### 2.4 Ethics

This study was conducted in accordance with the National Health Council's Resolution 466/12. IMIP's internal review board approved the study protocol (CAAE: 39928214.5.0000.5201). All women included in the prospective phase of the study signed an informed consent form.

## 3 Results

Fifty cases of acute PE were described: 38 were recovered retrospectively and 12 were followed-up prospectively. The mean age of the patients was 27.2 ± 8.1 years (±SD). Overall, 60% were from Recife or from other nearby towns comprising its greater metropolitan area, while 40% were from other towns in Pernambuco; 50.0% of the patients had 8 to 11 years of formal education, 22.0% >12 years and 28.0% <7 years of schooling (Table 1).

**Table 1:**
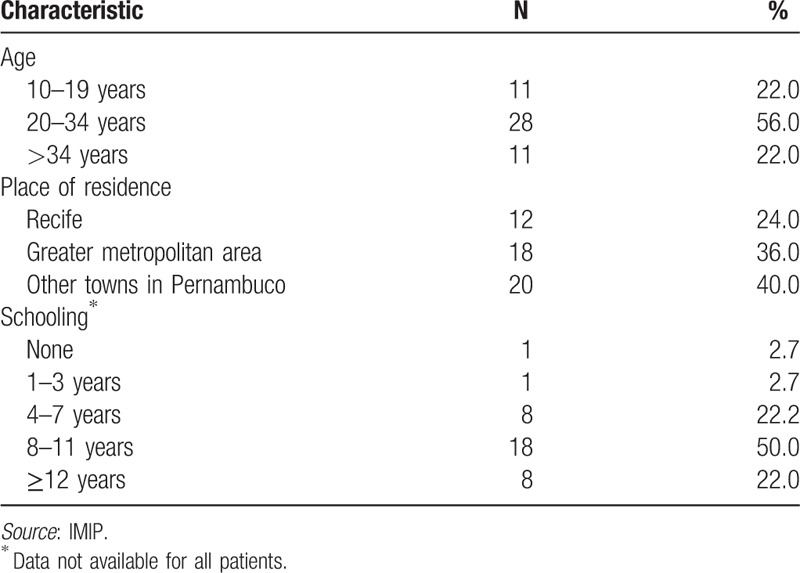
Patients’ sociodemographic characteristics.

The median number of pregnancies was 1 (interquartile range [IQR]: 1–3); 54.0% of the patients were primigravida mothers and 6% had multiple-gestation pregnancies (Table 2). Acute PE occurred antepartum in 58.0% of the patients, postpartum in 38.0%, and intrapartum in 4.0%. Of the patients analyzed, 8.0% were discharged from the ICU while still pregnant. Eight percent of patients had a history of prior acute PE, while 6.0% had a recurrence during hospitalization. Maternal mortality rate was 4.0%. Median time spent in the ICU was 5 days and median hospitalization time 11 days.

**Table 2:**
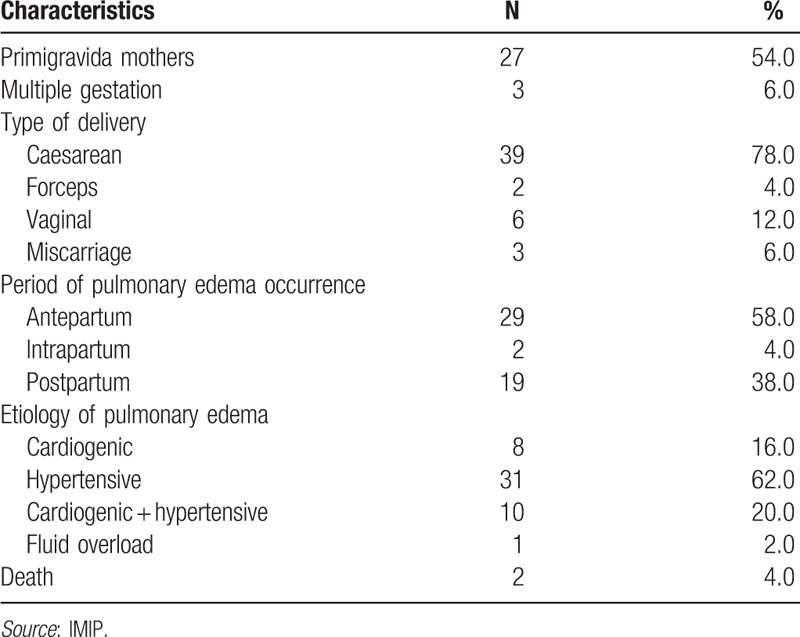
Patients’ obstetric characteristics.

The most common mode of delivery was Caesarean section (78.0%), with this rate being 42% in patients with acute PE antepartum and 34% in those whose condition developed postpartum. The most common type of anesthesia used was a spinal block (53.3%) or general anesthesia (22.2%) and 22.2% of deliveries occurred without anesthesia.

Hypertension alone was the etiology in 62% of cases: preeclampsia in 42%, eclampsia in 8%, superimposed preeclampsia in 10%, and chronic hypertension in 2%. Decompensated heart disease alone was the etiology in 16% of cases. A combination of hypertension and decompensated heart disease was the cause of acute PE in 20% of participants, while fluid overload was the single cause in 2% of cases.

Overall, 32.0% of patients received an intravenous infusion of fluids >2000 mL: 16% antepartum and 16.0% postpartum. The median positive fluid balance was 2225 mL (IQR: 1400–4400 mL). Oliguria was present in 12.0% of patients.

The principal signs and symptoms were: crackling rales (66.0%), dyspnea (64%), tachypnea (32.0%), coughing (20.0%), and wheezing (10.0%). Median systolic pressure was 160 mm Hg (IQR: 135–190 mm Hg), while median diastolic pressure was 96 mm Hg (IQR: 86.5–106.5 mm Hg). Median heart rate was 120 beats per minute (IQR: 100–130 bpm) and median respiratory rate was 32 breaths/minute (IQR: 24–39 breaths/minute). Median oxygen saturation was 91% (IQR: 85–95%).

Median time between diagnosis and initiating resuscitation maneuvers was 5 minutes, with maneuvers having already begun within 30 minutes of diagnosis in 75.0% of patients. All patients required ventilatory support, with the most common being the Venturi mask, used in 74% of cases. The rate of mechanical ventilation was 26.0%, and, of these cases, noninvasive mechanical ventilation was used in 30.0%. Median time of oxygen therapy was 36.0 hours (IQR: 27.0–72.0 hours).

*Near-miss* criteria were present in 60.0% of patients, with 52.0% having up to 3 criteria. Respiratory disorders were the most common: hypoxemia in 32.0% and intubation and ventilation unrelated to anesthesia in 26.0%. Vasoactive drugs were required by 20.0% of patients, and oliguria unresponsive to fluids or diuretics was recorded in 14.0% of the sample.

Transthoracic echocardiography was performed in 92.0% of the patients, either in their hospital bed or as outpatients. In the group of cases of cardiac-related acute PE, 12% of the patients had rheumatic heart disease, 2% had peripartum cardiomyopathy (PPCM), and 2% had Takotsubo cardiomyopathy. In the hypertensive group, echocardiography was performed in 74% of cases, with results showing that 20.0% of patients had heart failure or associated heart disease in addition to hypertension (14.0% had PPCM, while 6.0% had rheumatic heart disease).

Table 3 shows the echocardiographic findings according to the etiology of acute PE: cardiogenic, noncardiogenic, and both hypertensive and cardiogenic. The parameters analyzed were ejection fraction, left ventricular end-diastolic diameter, left ventricular end-systolic diameter, size of the left atrium, left atrial volume, tricuspid annular plane systolic excursion, and pulmonary artery systolic pressure. Some degree of ventricular dysfunction was detected in cases of cardiogenic and both cardiogenic and hypertensive etiology but not in the noncardiogenic group.

**Table 3:**
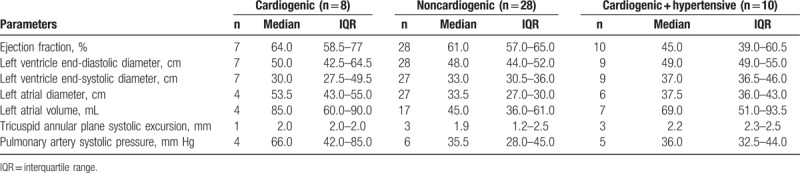
Echocardiographic findings in patients with acute pulmonary edema by etiology group.

Prematurity was very common, with 73.3% of deliveries occurring before 37 weeks of pregnancy and 39.0% occurring before 34 weeks (n = 45). Of the 50 patients studied, data were available for 51 fetuses. Intrauterine death occurred in 9.8% of cases, with death occurring after 24 weeks of pregnancy in 5.8% of cases. First and fifth minute Apgar scores were <7 in 63.8% and 3.4% of infants, respectively (data available for 47 infants).

Of the liveborn infants, 12.7% required resuscitation in the delivery room, with 17.0% requiring noninvasive ventilation and 10.6% needing orotracheal intubation. The most common comorbidity was respiratory disorders, present in 51.0% of cases, followed by jaundice (38.2%) and mild-to-moderate perinatal asphyxia (16.9%). Admission to the neonatal ICU was necessary in 25.5% of cases and neonatal death occurred in 7.8%.

## 4 Discussion

Acute PE is a major cause of maternal/perinatal morbidity/mortality. The principal etiology of the condition was hypertensive; nevertheless, echocardiography results showed that a significant proportion of these patients had associated cardiac disease, highlighting the importance of using this additional diagnostic tool to investigate the etiology of acute PE.

### 4.1 Comparison with other studies

The association between preeclampsia and acute PE has already been well-established, which explains the present finding of acute PE being mainly of hypertensive etiology. Therefore, not only the patient's hemodynamic status has to be taken into consideration, but also the poor fluid distribution resulting from the hypoproteinemia caused by the disease.^[1,3,8,9,13,14]^

The mean age of patients was 27.2 ± 8.1 years (±SD), which is similar to that reported by another study.^[1]^ Regarding the other clinical and demographic data, to the best of our knowledge no other studies have reported on these aspects.

Acute PE occurred most commonly before delivery (58.0%) or postpartum (38.0%). Similar rates were reported in a cohort study conducted in the United States with 51 cases of acute PE.^[1]^ This predisposition in pregnancy is probably due to cardiovascular changes such as increased plasma volume and cardiac output associated with reduced colloid osmotic pressure.^[3]^

Overall, 78.0% of deliveries were by Caesarean section, with acute PE being the reason for interrupting pregnancy and performing this procedure in 36.0% of cases. Due to the severity of the patient's state and her cervical conditions, it is often impossible to wait for labor to proceed naturally or even to induce labor, thus explaining the frequency of Caesarean sections.^[15]^

In 34.0% of cases in this study, acute PE developed following Caesarean section. In addition to fluid overload, which appears to trigger the onset of PE, the puerperium itself seems to increase vulnerability due to the postpartum autotransfusion phenomenon immediately following delivery, increasing cardiac output by up to 20% and reducing myocardial contractility.^[3]^

Almost all the patients requiring a Caesarean section had a spinal block. Although no association has been found between the type of anesthesia and acute PE, the infusion of fluids during anesthesia induction has been highlighted as a triggering factor.^[16]^ Fluid overload associated with acute PE was present in 34% of patients, with 16% consisting of patients who developed acute PE following Caesarean section. Fluid balance >2000 mL from intravenous fluids to increase plasma volume or treat oliguria is a known risk factor for acute PE.^[1,8,12,17]^

This study was unable to evaluate the hypothesis that oliguria would explain fluid overload. Only 10% of the patients with acute PE of hypertensive etiology and 2% of cases of both cardiogenic and hypertensive etiology had oliguria. Therefore, data on fluid balance may not have been precise, since the patients were not always rigidly controlled with respect to diuresis in the 24 hours preceding the onset of acute PE, either because diuresis was spontaneous or because diagnosis of acute PE had been made in another healthcare service. This situation may have led to a potential failure to observe a true association, a limitation that was also reported in another study.^[17]^ The conflicting findings in the literature may be related to the adoption of different approaches. At this institute, a regimen of restrictive fluid administration was established, both intrapartum and postpartum, significantly reducing the frequency of acute postpartum PE.

In a paper published in 1998, Mantel considered acute PE in itself a *near-miss* criterion.^[18]^ Nevertheless, in the 2010 WHO guidelines, used in the present study, the criteria were amplified and classified according to organ dysfunction.^[12]^ The most common types of *near-miss* in the present study were those associated with respiratory dysfunction related to hemodynamic instability and kidney failure. There are no studies correlating the natural history of acute PE with *near-miss* obstetric complications.

Detecting underlying heart disease in patients with acute PE is crucial.^[1,8]^ In the present study, patients diagnosed with heart valve disease (18%) were unaware that they had it. All the patients with structural disease diagnosed during the episode of acute PE had rheumatic heart disease, the most common in pregnancy.^[19]^

In the cases of acute PE of cardiogenic and mixed (both cardiogenic and hypertensive) etiology, echocardiography detected some degree of ventricular dysfunction. Conversely, when the etiology was noncardiogenic, echocardiogram results showed no evidence of this dysfunction and were, in the present sample, caused predominantly by preeclampsia (52%). These findings contradict the results of a case–control study of PE in preeclampsia.^[17]^ However, the small sample size must be mentioned as a limitation of that study.

Overall, 16% of the patients were diagnosed with pulmonary arterial hypertension (considered severe in 4% of cases). Heart failure was present in 16%, all of whom had a diagnosis of PPCM: 14% in association with a hypertensive disorder and only 2% with no other associated condition. Studies have called attention to the incidence of PPCM in patients with acute PE.^[16,19]^

Echocardiography is able to distinguish between acute PE of cardiogenic and noncardiogenic origin, as established in a case–control study conducted to evaluate acute PE in patients with preeclampsia.^[17]^ In that study, 92% of the patients were submitted to echocardiography, which contributed to the adequate diagnosis and treatment of the primary cause and to changing patients’ prognosis. An earlier study had already highlighted the benefits of echocardiography in obstetrics.^[8]^

Acute PE does not appear to have been the direct cause of any neonatal morbidity; however, it was an important indication for precipitating labor, thus exposing the newborn infants to the effects of prematurity and resulting in an increase in perinatal/neonatal morbidity/mortality. The high incidence of prematurity increased the need for neonatal resuscitation in the delivery room, required by 12.7% of the infants. Respiratory disorders were the most common complications, affecting half the infants. Respiratory problems constitute a common comorbidity in newborn infants unable to adapt rapidly to extra-uterine life, particularly premature infants.^[20]^ Due to lung immaturity, some infants may require oxygen supplementation, and the most common forms used in this study were noninvasive ventilation and orotracheal intubation, as recommended.^[21]^

### 4.2 Limitations of the study

Some limitations of this study include its retrospective design, with some potentially useful data being incomplete or unavailable. Since acute PE is a rare event, the sample was restricted to a stratified analysis, with limitations insofar as any more robust conclusions are concerned. New prospective studies with larger sample sizes should be conducted to identify factors associated with acute PE and its resulting mortality, including the possibility of using multivariate analysis to determine the adjusted risk.

The sample size was small but significant, principally compared to other previously published studies on the subject. Furthermore, the high percentage of cases in which echocardiography was performed, enabling the etiology of the condition to be defined in most cases, was a strongpoint of the study, adding to available knowledge on the disease.

## 5 Conclusions and policy implications

A protocol for the diagnosis and treatment of acute PE should include routine echocardiography at diagnosis due to its important role in identifying the specific etiology involved, particularly in this setting. Compliance with recovery care protocols accelerates clinical improvement and reduces hospitalization time.

## Author contributions

**Conceptualization:** Ana Carolina B. Pordeus, Leila Katz.

**Formal analysis:** Ana Carolina B. Pordeus, Leila Katz, Sabina B. Maia.

**Investigation:** Ana Carolina B. Pordeus, Mariana C. Soares, Sabina B. Maia.

**Methodology:** Leila Katz, Mariana C. Soares, Sabina B. Maia.

**Project administration:** Melania M. R. Amorim.

**Supervision:** Leila Katz, Melania M. R. Amorim.

**Writing – original draft:** Ana Carolina B. Pordeus.

**Writing – review & editing:** Sabina B. Maia, Melania M. R. Amorim.
